# Horizontal Cell Feedback to Cone Photoreceptors in Mammalian Retina: Novel Insights From the GABA-pH Hybrid Model

**DOI:** 10.3389/fncel.2020.595064

**Published:** 2020-11-04

**Authors:** Steven Barnes, James C. R. Grove, Cyrus F. McHugh, Arlene A. Hirano, Nicholas C. Brecha

**Affiliations:** ^1^Doheny Eye Institute, Los Angeles, CA, United States; ^2^Department of Ophthalmology, David Geffen School of Medicine, University of California, Los Angeles, Los Angeles, CA, United States; ^3^Department of Neurobiology, David Geffen School of Medicine, University of California, Los Angeles, Los Angeles, CA, United States; ^4^Neuroscience Graduate Program, University of California, San Francisco, San Francisco, CA, United States; ^5^Veterans Administration Greater Los Angeles Healthcare System, Los Angeles, CA, United States; ^6^Department of Medicine, David Geffen School of Medicine, University of California, Los Angeles, Los Angeles, CA, United States; ^7^Stein Eye Institute, David Geffen School of Medicine, University of California, Los Angeles, Los Angeles, CA, United States

**Keywords:** GABA receptor, horizontal cell, inhbitory feedback, photoreceptors, pH, center-surround inhibition, rho subunit, bicarbonate

## Abstract

How neurons in the eye feed signals back to photoreceptors to optimize sensitivity to patterns of light appears to be mediated by one or more unconventional mechanisms. Via these mechanisms, horizontal cells control photoreceptor synaptic gain and enhance key aspects of temporal and spatial center-surround receptive field antagonism. After the transduction of light energy into an electrical signal in photoreceptors, the next key task in visual processing is the transmission of an optimized signal to the follower neurons in the retina. For this to happen, the release of the excitatory neurotransmitter glutamate from photoreceptors is carefully regulated via horizontal cell feedback, which acts as a thermostat to keep the synaptic transmission in an optimal range during changes to light patterns and intensities. Novel findings of a recently described model that casts a classical neurotransmitter system together with ion transport mechanisms to adjust the alkaline milieu outside the synapse are reviewed. This novel inter-neuronal messaging system carries feedback signals using two separate, but interwoven regulated systems. The complex interplay between these two signaling modalities, creating synaptic modulation-at-a-distance, has obscured it’s being defined. The foundations of our understanding of the feedback mechanism from horizontal cells to photoreceptors have been long established: Horizontal cells have broad receptive fields, suitable for providing surround inhibition, their membrane potential, a function of stimulus intensity and size, regulates inhibition of photoreceptor voltage-gated Ca^2+^ channels, and strong artificial pH buffering eliminates this action. This review compares and contrasts models of how these foundations are linked, focusing on a recent report in mammals that shows tonic horizontal cell release of GABA activating Cl^−^ and HCO_3_^−^ permeable GABA autoreceptors. The membrane potential of horizontal cells provides the driving force for GABAR-mediated HCO_3_^−^ efflux, alkalinizing the cleft when horizontal cells are hyperpolarized by light or adding to their depolarization in darkness and contributing to cleft acidification *via* NHE-mediated H^+^ efflux. This model challenges interpretations of earlier studies that were considered to rule out a role for GABA in feedback to cones.

## What Is Feedback to Photoreceptors?

Output signaling from photoreceptors takes place at synaptic complexes comprising the photoreceptor terminal, horizontal cell synaptic processes, and bipolar cell dendrites, where visual information transfer and processing is initiated (Thoreson and Mangel, 2012). Here, essential aspects of visual processing, including center-surround antagonistic receptive field formation, color opponency, and sensitivity to spatiotemporal change, rely on lateral inhibitory feedback to photoreceptors by horizontal cells (Baylor et al., 1971; Mangel, 1991; Burkhardt, 1993; Dacey et al., 2000; Twig et al., 2003). This feedback was recently characterized as “The Case of the Missing Neurotransmitter” (Kramer and Davenport, 2015), emphasizing that the mechanisms proposed to underlie this feedback neurotransmission are not simple, fully characterized, agreed upon, or well understood (Thoreson and Mangel, 2012).

The *known common* targets of horizontal cell feedback reported in virtually all vertebrate species are the voltage-gated Ca^2+^ (Ca_V_) channels in the photoreceptor synaptic terminal (Verweij et al., 1996, 2003; Hirasawa and Kaneko, 2003; Vessey et al., 2005; Cadetti and Thoreson, 2006). These channels are necessary for photoreceptors to release neurotransmitters in the same manner that Ca_V_ channels are necessary for release in most other neurons, where presynaptic depolarization activates Ca_V_ channels, and this increases calcium influx that facilitates the release of neurotransmitter. However, photoreceptors hyperpolarize in response to light, meaning that during a light stimulus, the Ca_V_ channels become less activated, glutamate release decreases, and postsynaptic horizontal cells hyperpolarize. Since the horizontal cells extend lateral processes broadly, they receive input from a large number of photoreceptors, and they hyperpolarize strongly to a spatially large light stimulus but produce only a small hyperpolarization to a small spot of light.

**Figure 1:**
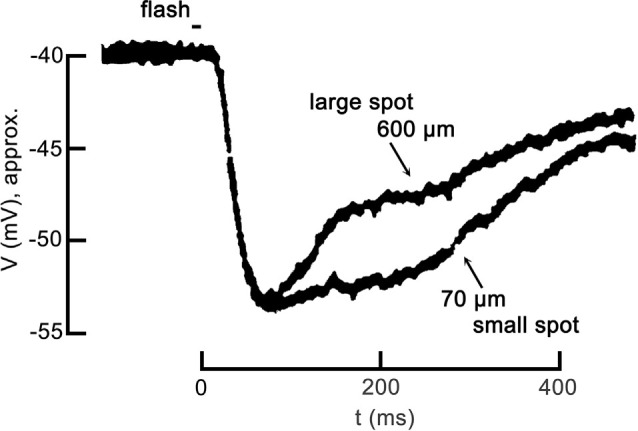
Center-surround antagonism in a cone photoreceptor due to feedback. The voltage response of a turtle cone to a small spot of light (70 μm diameter) and the response to a large spot (600 μm diameter) follow identical hyperpolarizing trajectories at the onset, but diverge during recovery. Both responses are plotted relative to the dark resting membrane potential of about −40 mV. The large spot response undergoes a pronounced inhibitory phase starting about 100 ms post-stimulus, becoming relatively more depolarized more quickly than the small spot response. The large spot recruited the receptive field surround of the cone via the large broad field of a horizontal cell, which feedback to the cone antagonizing the center response. Figure modified from Baylor et al. (1971).

Partial inhibition of cone Ca_V_ channel activation is the base functional state in darkness. To appreciate how the inhibition changes in response to patterned light stimulation, we describe the steps in the photoreceptor response to light, including Ca_V_ channel disinhibition during the response to a large spatial stimulus. In response to a brief, small spot of light, the cone hyperpolarizes, as seen in Figure 1. This is due to the light-induced closure of cGMP-gated channels in the photoreceptor outer segment, resulting in a reduction of the depolarization produced by those non-selective cation channels, allowing the standing K^+^ channel currents (I_Kx_) to hyperpolarize the cell, typically from about −40 mV to as much as −60 mV. However, in response to a large spot of light, an identical hyperpolarization occurs initially but this is followed by *inhibitory feedback* from strongly hyperpolarized horizontal cells that produce a delayed depolarizing phase in the cone response. Confusing as it may seem, this depolarization is what was originally referred to as *inhibitory feedback* (since it was an inhibition of the hyperpolarizing response to light), but we currently recognize that the underlying mechanism is a *disinhibition* of the cone Ca_V_ channels.

In this review, we discuss feedback in terms of the mechanism at the level of the photoreceptor Ca_V_ channels, and we refer to the terms “inhibition” and “disinhibition” in the context of horizontal cell modulation of photoreceptor voltage-gated Ca_V_ channels. For example, *inhibition* of cone Ca_V_ channels occurs when horizontal cells are depolarized during steady-state conditions of darkness or low light. In earlier reports, before the role of cone Ca_V_ channels in feedback was established by Verweij et al. (1996); “inhibitory feedback” was (and remains in many reports) terminology referring not to cone Ca_V_ channels but to the delayed depolarization that occurs in response to added surround illumination, which reduces (or “inhibits”) the hyperpolarizing response to focal stimulation of a cone with light (Figure 1). However, we now appreciate that since surround illumination produces strong *horizontal cell* hyperpolarization, this phenomenon observed in the cone membrane potential is caused by a *de facto* disinhibition of the cone Ca_V_ channels.

There is evidence for several feedback mechanisms, the most prominent of which include neurotransmitter-mediated signaling via GABA (or another/additional transmitter), interstitial current-induced external voltage changes, or “ephaptic coupling,” and pH-mediated surface charge screening effects. While similar descriptions of feedback from horizontal cells to photoreceptors have been reported in the retinas of non-mammalian vertebrates, the fewer reports in mammalian species have to account for significant underlying mechanistic differences from non-mammalian species (Thoreson and Mangel, 2012; Liu et al., 2013). These differences between mammalian and non-mammalian species are important to note, as they have previously complicated attempts to understand this synaptic feedback circuit (Wu, 2010; Thoreson and Mangel, 2012; Liu et al., 2013; Kramer and Davenport, 2015).

## Mechanisms of Feedback to Photoreceptors

### GABA

The early detection of GABA in horizontal cells (Lam et al., 1978) propelled investigation into how horizontal cell release of GABA would mediate feedback. At the time, models presumed a simple inhibitory neural action and it seemed safe to assume that horizontal cells would release GABA when they are depolarized and that the released GABA would bind to presumptive GABARs on photoreceptors, increasing Cl^−^ conductance, leading to photoreceptor hyperpolarization. Even then such a mechanism for GABA was difficult to reconcile since, as a starting point, either the hyperpolarized horizontal cell had to release a transmitter that produced a sign-inverting depolarization in the cone associated with a conductance increase, or the horizontal cell had to release a neurotransmitter that decreased conductance in cones in the dark, and when reduced by light, this led to increased cone conductance (Baylor et al., 1971). Later reports continued to support a role for horizontal cell release of GABA (Cueva et al., 2002; Hirano et al., 2005, 2016; Guo et al., 2010). In mammalian retinas, compelling evidence suggests that horizontal cells release GABA in a depolarization-dependent, vesicular manner (Hirano et al., 2016; Grove et al., 2019). In mammals, GABA and the GABA synthetic enzyme, L-glutamate decarboxylase (GAD) are localized to horizontal cells (Schnitzer and Rusoff, 1984; Wässle and Chun, 1989; Grünert and Wässle, 1990; Vardi et al., 1994; Guo et al., 2010; Schubert et al., 2010; Deniz et al., 2011) and VGAT, V-ATPase, multiple SNARE, and vesicle proteins, and Ca_V_ channels mediating vesicular release are localized to horizontal cell dendritic tips and axonal terminals (Dowling and Boycott, 1966; Brandon and Lam, 1983; Linberg and Fisher, 1988; Peters et al., 1991; Catsicas et al., 1992; Ueda et al., 1992; Löhrke and Hofmann, 1994; Grabs et al., 1996; Greenlee et al., 2001; Rivera et al., 2001; Cueva et al., 2002; Hirano et al., 2005, 2007, 2011; Schubert et al., 2006; Lee and Brecha, 2010; Liu et al., 2013). Furthermore, vesicle membrane fusion and recycling in horizontal cells is depolarization—and Ca^2+^-dependent (Takamori et al., 2000; Vuong et al., 2011), and the deletion of VGAT from horizontal cells abolishes horizontal cell inhibitory feedback to photoreceptor Ca_V_ channels (Hirano et al., 2016). This evidence leads to the conclusion that depolarization-mediated, Ca^2+^-dependent GABA release could mediate horizontal cell signaling. However, due to several additional observations, including the low concentration of vesicles in horizontal cells synaptic terminals, and the persistent GABA presence around horizontal cell synaptic endings, it has been suggested that GABA release could be continuous, increasing with depolarization, but slowly and with limited uptake and degradation in the synaptic cleft (Grove et al., 2019).

The existence of many similar features of feedback in mammalian and non-mammalian vertebrates suggests conservation of mechanisms but may serve as false flags when making comparisons, and there are critical differences concerning GABA. (1) Non-mammalian vertebrates have not been proven to have a GABA vesicular release mechanism in horizontal cells similar to that in mammals. Instead, early discoveries in several non-mammalian vertebrates concluded that reversed GABA-uptake transporters in horizontal cells mediate the release of GABA (Schwartz, 1982, 1987, 2002; Yazulla and Kleinschmidt, 1983; Ayoub and Lam, 1984). Mammalian horizontal cells do not have GABA-uptake transporters (GATs; Johnson et al., 1996; Guo et al., 2009, 2010), so they do not emulate this mechanism of action. (2) While non-mammalian vertebrates also appear to have GABA in some of their horizontal cells, many have been reported to not contain GABA and some may employ different neurotransmitters. This said, there are important similarities. Both types of vertebrates have GABA receptors on their horizontal cells and both have elevated chloride equilibrium potentials (around −30 mV). These similarities suggest that vertebrates may employ the same GABA-pH hybrid mechanism, albeit with a different means of GABA release, the details of which are discussed extensively in the body of this review.

In non-mammalian species, horizontal cell release of GABA appears to directly hyperpolarize photoreceptors (Skrzypek and Werblin, 1983; Tachibana and Kaneko, 1986; Wu, 1991, 1994; Tatsukawa et al., 2005; Endeman et al., 2012). In contrast, a direct action of GABA on photoreceptors has not been unequivocally established in mammals. Many physiological recordings in the normal mammalian retina do not show a direct action of GABAergic agents on cones (Verweij et al., 2003; Crook et al., 2009; Kemmler et al., 2014; Szikra et al., 2014; Grove et al., 2019), but there is evidence for GABAR subunit expression by mammalian photoreceptors (Greferath et al., 1993; Grigorenko and Yeh, 1994; Picaud et al., 1998; Vardi et al., 1998; Chaffiol et al., 2017). However, in *cultured* retinal explants (and possibly in rd1 mice lacking rods), cones may be reprogrammed and respond to GABA application (Picaud et al., 1998; Pattnaik et al., 2000), and one report indicates GABA activation of TPMPA-insensitive GABAR Cl^−^ channels in wild-type mouse cones (Deniz et al., 2019). Further complicating the functional role for photoreceptor Cl^−^ flux, during surround light stimulation that hyperpolarizes horizontal cells, the resulting disinhibition of Ca_V_ channels in cones in fish (Verweij et al., 1996) and *macaque* (Verweij et al., 2003) is accompanied by an increase in Ca^2+^-activated Cl^−^ conductance. These events are not easily reconcilable with a direct ionotropic GABA action in cones and would be more confidently considered to be due to a reduction of Cl^−^ conductance during reduced GABA release by horizontal cells.

An additional long-standing controversial issue was that GABA release by depolarized horizontal cells directly mediating feedback to cones required an atypical mechanism. Baylor et al. (1971) suggested that the inhibition of the light response defied any known neurotransmitter mechanism. Horizontal cell hyperpolarization leads to cone depolarization, which is associated with a conductance increase. A solution, reviewed here, is that horizontal cell feedback signaling is mediated by GABA acting *indirectly* on photoreceptors. This is supported by findings that GABA acts autaptically on horizontal cells, whose depolarization and membrane properties result in pH-regulated inhibition of photoreceptor Ca_V_ channels (Liu et al., 2013; Grove et al., 2019).

Since horizontal cell feedback affects cone synaptic output to bipolar cells, horizontal cell influences carry through to these cells. Also, horizontal cells appear to inhibit directly the dendrites of many types of bipolar cells. Horizontal cell signaling mediated by GABA to bipolar cells is consistent with GABARs on bipolar cell dendrites (Vardi et al., 1992; Enz et al., 1996; Wässle et al., 1998; Haverkamp and Wässle, 2000; Haverkamp et al., 2000; Hoon et al., 2015), although which bipolar cell types and their complement of dendritic GABAR subtypes are unknown (Chaffiol et al., 2017). Different [Cl^−^]_i_ levels in bipolar cell dendrites, maintained by two types of chloride co-transporters (KCC2 and NKCC; Vardi et al., 2000; Vu et al., 2000) could account for a direct inhibitory and excitatory effect caused by GABA released by horizontal cells at ON- and OFF-bipolar cells (Miller and Dacheux, 1983; Satoh et al., 2001; Varela et al., 2005; Duebel et al., 2006). Both feedback via cones and direct feedforward signaling pathways have a strong influence on bipolar cell responsiveness and all downstream neurons in the retina and visual system.

### Ephaptic Coupling and the Role of Hemichannels

The unique, enveloping structural constraints of this synapse, where horizontal cell synaptic endings invaginate rod and cone presynaptic terminals led to the proposal of the “electric feedback model” as the mechanism of feedback (Byzov, 1977; Byzov et al., 1977; Byzov and Shura-Bura, 1986). This model was based on the fact that current flow through a resistive medium (here, the tortuous extracellular paths through which current flows to enter glutamate receptor channels in the synaptic endings), constitutes a resistance, which according to Ohm’s law creates a voltage drop at the horizontal cell synaptic tips. The result is a net negative extracellular voltage in the synaptic cleft relative to ground (0 mV). By producing an external negative potential here, outside the cone membrane at the synaptic release site, the electric field across the membrane of the adjacent cone is reduced, affecting equivalent to depolarization of the cone membrane that increases the activation of the photoreceptor Ca_V_ channels responsible for glutamate release (Taylor and Morgans, 1998; Nachman-Clewner et al., 1999; Morgans, 2001). The physics of this model is solid, but there is a lack of certainty of the amplitude of the external voltage drop, and more troubling for the model, when the horizontal cell glutamate-activated postsynaptic current is reduced during a strong light stimulus, hyperpolarizing the cell, interstitial current flow to the synapse is reduced, and feedback modulation is diminished or even vanishes.

Decades later, Kamermans et al. (2001) solved this dilemma, upgrading Byzov’s model (Byzov, 1977; Byzov et al., 1977; Byzov and Shura-Bura, 1986) by incorporating the finding that hemichannels, each half of a gap junction channel and composed of connexin26, had been identified at the tips of fish horizontal cell dendrites deep within the invagination (Janssen-Bienhold et al., 2001a,b). This clever improvement circumvented the perceived problems caused by the closure of glutamate-gated channels by invoking the presence of ion channels that were not gated by glutamate and that would reliably produce the interstitial current flow required for continuous extracellular non-zero voltage modulation. According to this new hemichannel hypothesis, surround illumination that causes strong horizontal cell hyperpolarization and greater inward current through hemichannels in their synaptic endings (Kamermans et al., 2001; Fahrenfort et al., 2005), producing a voltage drop in the synaptic cleft. While interstitial voltage cannot be reliably measured, this action is revealed in voltage-clamped cones during surround illumination, acting as a shift in the activation curve of the cone Ca_V_ channel current to more positive potentials (Verweij et al., 1996, 2003) increasing glutamate release, this being the feedback that opposes the cone hyperpolarization.

Hemichannels at the photoreceptor synapse were found in goldfish, zebrafish, and turtles on the membranes of the lateral processes of horizontal cell tips, deep within the synaptic terminal (Kamermans et al., 2001; Pottek et al., 2003; Klaassen et al., 2011). Pharmacological blockade of hemichannels with carbenoxolone blocked feedback-mediated responses in non-mammalian cones and horizontal cells (Kamermans et al., 2001). According to the model, by blocking hemichannels, carbenoxolone restores the suppression of cone Ca_V_ channels through an apparent rightward shift of the activation curve, reducing the amount of glutamate is released. It should be noted that while carbenoxolone has been widely used as a functional probe for gap junctions, this diagnostic tool depends entirely on the specificity of its actions, and there are reports it can act on multiple targets. In addition to blocking gap junctions, carbenoxolone has been shown to suppress action potentials, decrease input resistance, block Ca_V_ channels, block postsynaptic NMDA receptors, and reduce inhibitory synaptic currents through a direct effect on GABARs (Rekling et al., 2000; Rouach et al., 2003; Vessey et al., 2004; Tovar et al., 2009; Beaumont and Maccaferri, 2011; Connors, 2012). Thus, the effects of carbenoxolone do not constitute conclusive evidence that gap junctions are involved, especially when GABARs and Ca_V_ channels are involved.

The hemichannel model was bolstered by comparing normal and genetically modified zebrafish that lack connexin hemichannels in horizontal cells (Klaassen et al., 2011). Feedback was reduced in the mutants, supporting the hemichannel role in feedback from horizontal cells to cones. Intracellular recordings in horizontal cells showed color-opponent responses were diminished and the mutant fish also showed decreased contrast sensitivity in behavioral tests, expanding the reach of the model to the functional level in visual processing.

The role of hemichannels in horizontal cell feedback in zebrafish was further expanded to include pH effects at the synapse. In addition to connexin hemichannels mediating rapid feedback actions, pannexin hemichannels are implicated in ATP release, which induces extracellular acidification through hydrolysis of ATP by endonucleotidases in the cleft (Kurtenbach et al., 2014; Vroman et al., 2014). Pannexin/ATP-mediated feedback is a Ca_V_ channel inhibiting mechanism occurring with depolarization of the horizontal cell, while connexin-mediated feedback produces disinhibition when horizontal cells are hyperpolarized.

Physics sets the time course of hemichannel mediated ephaptic feedback, and it must occur instantly in response to changes in current flow in the glutamate-gated or hemichannel conductances, which for both depends on the horizontal cell membrane potential, regulated primarily by changes in glutamate levels in the cleft. The speed of feedback signaling has been used as a diagnostic tool, but it remains difficult to discriminate between models due to the layering of their actions.

As is the case with GABA, there are many differences between mammalian and non-mammalian retinas concerning hemichannels. Connexin hemichannels are not found in mammalian (rodent) horizontal cell tips. Analysis of pannexins in mouse horizontal cells shows sparse localization away from the invaginating tips of the horizontal cells (Kranz et al., 2013).

### Photoreceptor Synapse Modulation by pH

**Figure 2:**
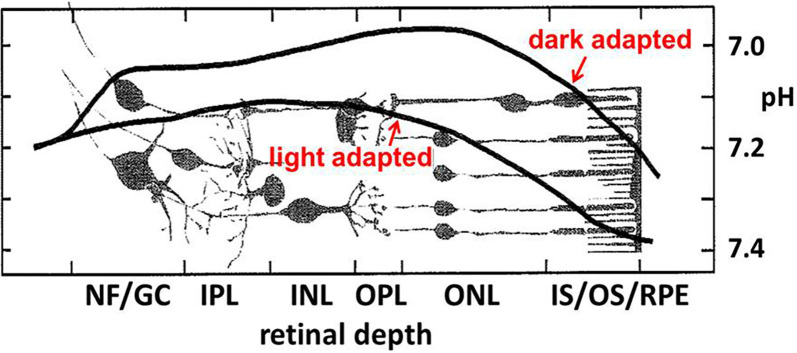
Near the photoreceptor layer, there is a notable light vs. dark difference in bulk pH_o_ in the outer retina. In the darkness, when rod and cone photoreceptors are maximally depolarized for an extended period, the pH becomes more acidic. Figure adapted from Yamamoto et al. (1992).

Over many decades an appreciation has emerged that extracellular pH (pH_o_) fluctuates in healthy brain tissues. Assessment of pH homeostasis in the vertebrate retina showed significant disparities from the earlier conception that pH_o_ was one of the best-regulated homeostatic variables necessary for brain function (Yamamoto et al., 1992; Dmitriev and Mangel, 2004; Dmitriev et al., 2016). Not only did retinal measurements of pH_o_ reveal values far from the pH 7.4 seen in the vasculature, but pH_o_ in the retina changed dramatically, depending on light stimulation, showing that the dynamic nature of pH_o_ in tissue with high energy consumption exceeds those in other nervous and somatic tissues. In the retina, pH_o_ is most acidic in the dark within the outer nuclear layer (ONL—composed of the cell bodies of rod and cone photoreceptors; Figure 2), with Müller cell processes. At the level of the retinal pigmented epithelium (RPE), pH increases, approaching that of the blood (pH 7.4) owing to the proximity of the choroidal supply. At the nerve fiber (NF) layer and ganglion cell (GC) bodies, pH also increases, presumably due to the voluminous and unimpeded buffering capacity provided by the aqueous humor, to a value near 7.2. What is more profound about this spatial disparity, is that under light-adapted conditions, the bulk pH increases at all layers across the retina, and the point of greatest change is the ONL, where the mitochondria of rods and cones are maximally active in the dark, and reduced in bright light, nearly completely in the case of rods at least, when they are hyperpolarized.

This spatiotemporal pattern of pH_o_ reflects energy use by the cells. For example, in mouse rods, to maintain tonic depolarization, a high energy demand exists for the removal of Na^+^ entering through CNG channels and Na^+^/(Ca^2+^+K^+^)-exchangers of the outer segment, and replenishment of K^+^ that effluxes via these and voltage-gated Kx channels of the inner segment, and pumping of Ca^2+^ from the synaptic terminal. ATP use in mouse rods is increased by a factor of 5 in the dark compared to bright light, with most of this due to increased Na^+^/K^+^-ATPase and Ca^2+^-ATPase activity (Krizaj and Copenhagen, 2002; Okawa et al., 2008). In general, when neurons are depolarized their ion channels are more frequently open (except some inward rectifier K and HCN channels) and their metabolic activity increases to maintain concentration gradients for Na^+^, K^+^, Ca^2+^, and other ions. The active transport of these ions by the Na^+^/K^+^-ATPase and plasma membrane Ca^2+^-ATPase is acknowledged to be the largest energy expenditure for neurons (Ames et al., 1992; Niven and Laughlin, 2008). At excitatory synapses where Na^+^ influx and K^+^ efflux can be protracted, and especially so in the present case where second-order neurons such as horizontal cells are in a tonically depolarized state during low illumination due to the continuous release of glutamate from photoreceptors, the energy cost of active ion transport to maintain transmembrane ion gradients are high (Wong-Riley, 2010). Thus, the energy requirements of the retina are higher in the dark than in the light, and in producing ATP, the neurons extrude H^+^ prodigiously, making pH_o_ low (Ames et al., 1992). It has long been appreciated that the high metabolic requirements and their dependence on illumination contribute to the sustained, low bulk pH in the outer retina in the dark and its increase during illumination (Borgula et al., 1989; Oakley and Wen, 1989; Yamamoto et al., 1992). Given this backdrop of pH_o_ in the outer retina, it is noteworthy that an additional mechanism underlying feedback inhibition involves the regulation of pH_o_ in the photoreceptor synaptic cleft (Thoreson and Mangel, 2012; Vroman et al., 2014; Wang et al., 2014; Beckwith-Cohen et al., 2019). Changes in pH_o_ provide powerful modulation of voltage-gated ion channels due to membrane surface charge effects. This biophysical action occurs due to protons interacting with the fixed negative surface charge of the bilayer and membrane proteins, altering the electric field sensed by the voltage-sensors of ion channel proteins present in the membrane, leading in the present case to reduced photoreceptor Ca_V_ activation (Hille, 1968; Barnes and Bui, 1991; Barnes et al., 1993). Increased pH buffering of the retina with Hepes suppresses feedback and concentrations as low as 10 mM are enough to reversibly block it (Barnes et al., 1993; Hirasawa and Kaneko, 2003; Vessey et al., 2005; Cadetti and Thoreson, 2006; Davenport et al., 2008; Thoreson et al., 2008; Fahrenfort et al., 2009; Liu et al., 2013).

**Figure 3:**
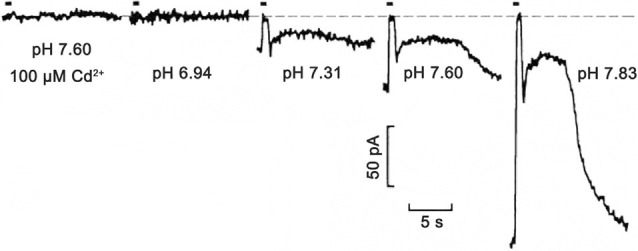
Extreme pH sensitivity of synaptic transmission from photoreceptors to horizontal cells. Light responses to 500 ms bright light steps measured in a salamander horizontal cell under voltage-clamp were fully blocked at pH 6.94, to the same extent as in the presence of Ca_V_ channel 100 μM Cd^2+^ at pH 7.6. Zero current is shown by the dashed line. As pH was increased to 7.31, 7.60, and 7.83, the postsynaptic, light-induced glutamate-gated current changes were produced by the hyperpolarizing cone light responses (in the figure, the short up-going currents aligned with light stimulus timing bar) and rod response (slower and longer upward deflections) become larger. This is due to the turnoff by the light of the increased glutamate release in the dark. The exponentially increasing response amplitudes at increasing pH levels were shown to be due to surface charge screening effects on the voltage-gated Ca_V_ channels in presynaptic photoreceptors. Figure modified from Barnes et al. (1993).

Shifts in synaptic cleft pH modulate the voltage dependence of photoreceptor Ca_V_ channel activation and this regulates glutamate release from photoreceptors (Barnes et al., 1993; Cadetti and Thoreson, 2006; Babai and Thoreson, 2009). This clear and simple relationship demonstrates how activity-driven changes in pH in the synaptic cleft can affect synaptic regulation (Figure 3). Whether adaptations to the expression of Na^+^/H^+^ exchangers (NHEs), HCO_3_^−^ transporters (NBCs, AEs, NCBEs, and NDCBEs), V-ATPases, monocarboxylic acid transporters (MCTs) and carbonic anhydrase (CA; Soto et al., 2018), have occurred in the outer retina to mitigate or potentiate the contribution of acidification to this feedback mechanism is not known.

As a final introductory remark, we highlight the remarkably potent effects that extracellular pH has on Ca_V_ channel activation and gating, which is shared in all tissues and all species (Barnes et al., 1993; Neumaier et al., 2015). Changes in extracellular pH produce changes in the voltage-dependent activation of ion channels, due to the change in proton concentration causing differing degrees of proton adsorption to the fixed negative surface charge of the lipid bilayer proteins and binding to exposed ion channel protein amino acid side groups. The extreme proximity of protons to the surface of the cell membrane alters the electric field sensed by the ion channel voltage sensors, in effect adjusting their activation point at a given membrane potential (Barnes et al., 1993). Increased positive surface charge adsorption, as produced by decreased pH_o_ (i.e., increased [H^+^]_o_) alters the electric field in the membrane acting upon the channel voltage sensor moves, producing the same action that a more negative membrane potential does, i.e., decreasing the probability of channel opening and moving the measured half-maximal activation voltage to more positive potentials. The relation between the pH and activation midpoint shift is on the order of a 1 pH unit decrease causing a 10 mV negative shift of V_½_ (Barnes et al., 1993). This means that a greater degree of membrane depolarization is required to activate ion channels under increasingly acidic extracellular pH’s. This is how synaptic cleft pH alterations at the photoreceptor output synapse can potently alter the postsynaptic signals in horizontal and bipolar cells.

Accommodating these foundations, a new model based on the specific properties of horizontal cells in the mammalian retina, including the now-established release of GABA by horizontal cells (Hirano et al., 2016; Grove et al., 2019), demonstrated that horizontal cell-released GABA acts back, autaptically, on horizontal cell GABA receptors, and due to their intrinsic permeability to [HCO_3_^−^], facilitates its efflux, which modulates photoreceptor transmitter release via pH changes in the synaptic cleft (Liu et al., 2013; Grove et al., 2019). Also, especially given the richness of investigations in non-mammalian species (Byzov and Shura-Bura, 1986; Verweij et al., 1996; Kamermans et al., 2001; Vessey et al., 2005; Jackman et al., 2011; Klaassen et al., 2011; Kramer and Davenport, 2015) there may be other pathways by which horizontal cells affect photoreceptors (Kemmler et al., 2014).

## Ca_V_ Channels in Mammalian Cones Are Tonically Modulated in a GABA- and pH-Dependent Manner

The targets of horizontal cell feedback in photoreceptors are the voltage-gated Ca_V_ channels that mediate glutamate release from the presynaptic terminals (Verweij et al., 1996, 2003; Hirasawa and Kaneko, 2003; Vessey et al., 2005; Cadetti and Thoreson, 2006; Montgelard et al., 2008; Thoreson et al., 2008). While different mechanisms of feedback may dominate: (1) in specific species; (2) under different conditions of ambient illumination; and (3) over distinct temporal domains, here we will examine mechanisms operating under mesopic conditions using mammalian (rodent) retinas under this steady-state lighting condition (Grove et al., 2019). Due to earlier work showing a role for GABA in feedback, and with vesicular GABA release by horizontal cells being perhaps the most obvious difference between mammalian and non-mammalian horizontal cells, we review evidence for the role of GABA in the inhibition of mammalian photoreceptor Ca_V_ channel activation. The component actions reviewed below allow dissection of the sequential mechanisms underlying features of feedback by probing the steady-state response of cones and horizontal cells, under constant conditions of illumination, as a baseline to identify mechanisms underlying feedback.

**Figure 4:**
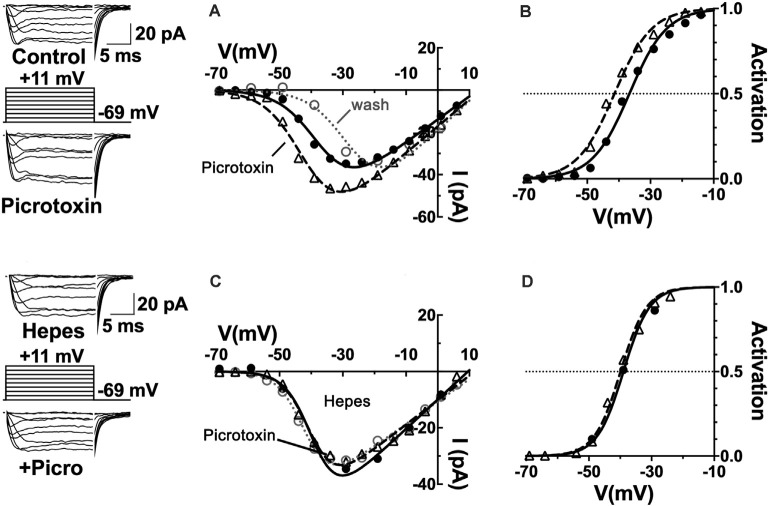
Ca_V_ channels in mammalian cone photoreceptors are maintained in a pH-mediated, tonically inhibited state by GABA receptor activation. **(A)** The GABA_A_R antagonist picrotoxin increased calcium current amplitude in cones, maintained in mesopic conditions. Sample current traces and I–V relations of a rat cone before (solid line, filled circles), during (dashed line, triangles), and after (dotted line, open circles) application of 100 μM picrotoxin to the retinal slice. **(B)** The Ca_V_ channel activation curve for the cell underwent a leftward shift in the half-maximal activation voltage in picrotoxin (midpoint = −41 mV) vs. control (midpoint = −37 mV). **(C)** Same experimental protocol as above applied while clamping superfusate pH to 7.4 with 10 mM Hepes. I–V relation in Hepes alone is shown (solid line, circles) and then with picrotoxin (dashed line, triangles). Washout superimposes (dotted line, open circles). **(D)** Hepes eliminated the effect of picrotoxin on Ca_V_ channel activation (midpoint = −39 mV). Figure modified from Grove et al. (2019).

First, to what extent are GABARs involved in the steady-state inhibition of cone Ca_V_ channels? The Ca_V_ channel currents of cones patch-clamped in retinal slices were found to be increased by about 60% in mice, 40% in rats, and 25% in guinea pigs when the non-competitive ionotropic GABAR antagonist picrotoxin was superfused at 100 μM. Figure 4 shows these results from rat retina, with the amplitude of Ca_V_ channel currents increased in the raw current traces, in response to voltage command steps, as well as in the current-voltage (I–V) relations made from these currents under the two conditions. When the I–V relations were divided by the driving force for Ca^2+^ and fit with a Boltzmann function to define the Ca_V_ channel activation curves, it was found that the half-maximal activation voltage shifted in mice, rats, and guinea pigs to a ~5 mV more negative voltage. A shift of the channel activation curve to more negative potentials in picrotoxin represents the disinhibition of cone Ca_V_ channel currents as channel open probability increases at physiological membrane potentials. These results imply that there is tonic GABA inhibition of cone Ca_V_ channels under mesopic conditions in these species.

The observation that horizontal cells inhibit photoreceptor output by shifting their Ca_V_ channel activation curves is consistent with earlier proposals (Verweij et al., 1996, 2003; Kamermans et al., 2001). The vital distinction is that these data show an unappreciated role for the transmitter GABA as well. The classical role of GABA in feedback, as shown through the antagonism by picrotoxin observed in fish cones (Endeman et al., 2012) involves direct activation of GABARs on cones, which is not an action seen in Figure 4, where no change in steady-state conductance of a Cl^−^ current was noted. Picrotoxin blocks the ion channel pore of ionotropic GABA_A_ receptors (Ashiya et al., 1995), as well as glycine receptors (Johnston, 2014), and does not affect Ca_V_ channels *per se*. It did not decrease standing cone conductance that would have been present if GABA had been activating photoreceptor Cl^−^ channels. No difference was observed in cone membrane conductance measured between −80 and −50 mV with and without picrotoxin in mice, rats, or guinea pigs, suggesting that the absence of a tonic, direct GABAergic input onto GABARs expressed in photoreceptors (Grove et al., 2019).

It bears mentioning that the results obtained in the fish retina were obtained using transient light flashes in a dark-adapted state, not the steady state mesopic conditions in Figure 4. The fact that the component action shown here is occluded when interstitial pH is clamped with the pH buffer Hepes (Figure 4), is interpreted as picrotoxin changing pH to modulate photoreceptor Ca_V_ channels. The pH sensitivity of horizontal cell feedback and photoreceptor Ca_V_ channels (Hirasawa and Kaneko, 2003; Vessey et al., 2005; Cadetti and Thoreson, 2006; Thoreson et al., 2008; Vroman et al., 2014; Warren et al., 2016a), together with reports in mammals regarding the actions of GABA antagonists in the rat (Liu et al., 2013), guinea pig, mouse (Grove et al., 2019) and *macaque* (Verweij et al., 2003), connect this action of GABA to the change of cleft pH.

Earlier suggestions that Hepes stifles feedback by acidifying the cytoplasm of all local neurons, and that due to that, the true mechanism of feedback cannot be sustained (Fahrenfort et al., 2009). This argument failed when results of numerous other pH-buffers (Davenport et al., 2008), as well as increased [HCO_3_^−^] (Vessey et al., 2005), were found to have the same effects on feedback, but without cytoplasmic acidification. A more recent theory that cleft pH changes are due instead to hydrolysis of ATP released via pannexin hemichannels (Vroman et al., 2014; Cenedese et al., 2017), is consistent with the effect of Hepes, however, the theory is mute in mammals since pannexins are not present at horizontal cell endings in the synaptic invagination (Kranz et al., 2013).

## What Type of GABA Receptors Are Responsible for This Unconventional Effect?

**Figure 5:**
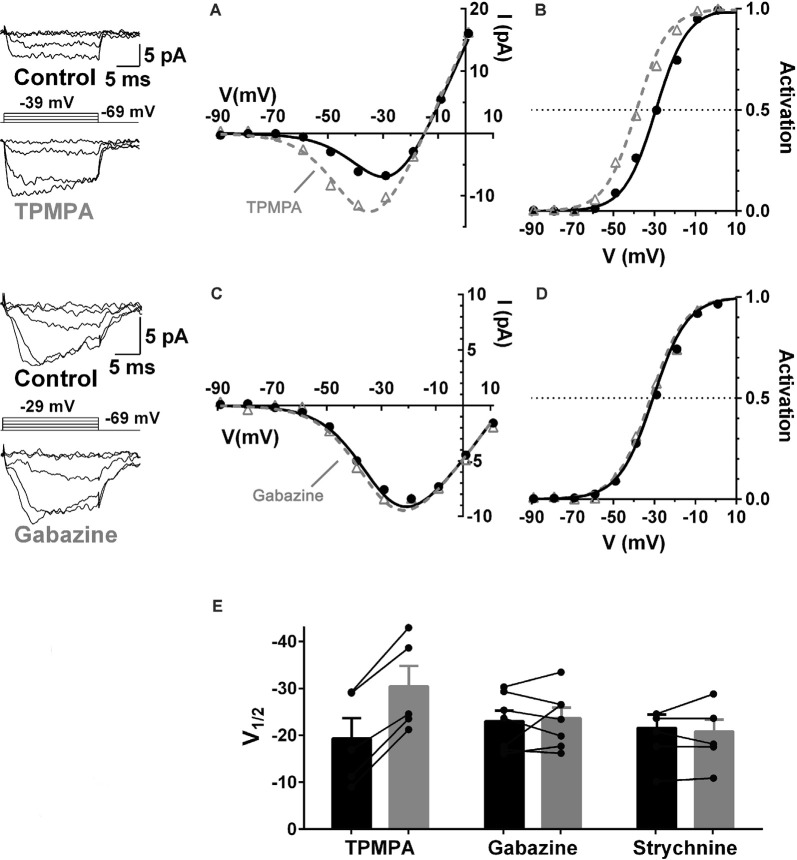
GABA receptors containing ρ-subunits mediate the modulation of cone Ca_V_ currents. Effects of GABAR blockers on Ca channel currents in cones in guinea pig retinal slices. **(A,B)** The ρ-subunit-containing GABAR antagonist TPMPA (50 μM) shifted Ca_V_ activation in guinea pig cones to more negative voltages, in this case by 10 mV. **(C,D)** The GABA_A_R antagonist gabazine (10 μM) did not affect Ca_V_ activation. **(E)** Summary of the effects of TPMPA, gabazine, and the GlyR antagonist strychnine (100 μM) on Ca_V_ channel activation (5–7 cones). In each pair the filled bar (on left) is control and the gray bar (on right) shows the change in the presence of the blocker. Figure modified from Grove et al. (2019).

GABA receptors containing ρ-subunits (a.k.a. GABA_C_Rs) appear to play the central role in the modulation of the cone Ca_V_ channel currents. The ρ-subunit-containing GABAR inhibitor TPMPA, the GABA_A_R inhibitor gabazine, and the glycine receptor inhibitor strychnine were tested for their ability to produce Ca_V_ channel activation curve shifts in cones, and only TPMPA produced activation shifts. This result identified ρ-subunit-containing GABA receptors as the mediators of this action of GABA. Figure 5 shows that the superfusion of guinea pig retinal slice with the ρ-subunit-containing GABA receptor antagonist TPMPA increased the cone Ca channel current amplitude. This was accompanied by a negative shift of the Ca_V_ channel activation curve with V_½_ decreasing by about 10 mV. TPMPA had a similar action on mouse cones, shifting the activation curve negative by about 6 mV. Neither gabazine nor strychnine produced activation curve shifts in rodents.

Similar to the effects of picrotoxin discussed in Figure 4, TPMPA, gabazine, and strychnine did not produce conductance decreases in cones between −90 and −60 mV, a zone well away from the Ca_V_ channel activation range, suggesting that GABARs are not functional in cones under the recording conditions used here.

## Where Are These ρ-Subunit-Containing GABARs Located?

The pharmacological results in Figure 5 indicated a role for GABA receptors containing ρ-subunits in feedback. ρ-subunit-containing GABA receptors are not found in mammalian cones but these GABARs have been identified in horizontal cells with GABA-mediated responses reported in fish, salamander, rat, guinea pig, and mouse horizontal cells (Wu and Dowling, 1980; Kamermans and Werblin, 1992; Dong et al., 1994; Takahashi et al., 1995; Verweij et al., 1998; Yang et al., 1999; Bormann, 2000; Feigenspan and Weiler, 2004; Liu et al., 2013; Grove et al., 2019).

**Figure 6:**
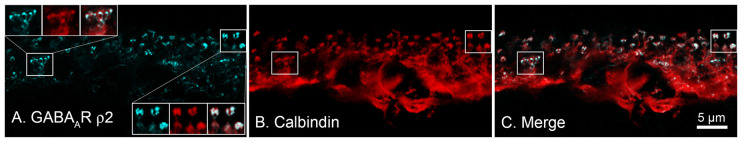
Horizontal cells express ρ-subunit-containing GABA receptors in their synaptic endings in the mouse retina. **(A)** Immunolocalization of the ρ2 subunit (blue). **(B)** Horizontal cell marker, calbindin immunoreactivity (red). **(C)** Merged image shows that expression of ρ2 subunits is limited to the tips of horizontal cell synaptic endings that invaginate rod and cone photoreceptor terminals (Insets show magnified images in Panel **(A)**, upper left, of a single cone pedicle; lower right, 3-rod spherules). Figure modified from Grove et al. (2019).

Concerning data supporting a role for this subset of GABARs in modulating cone Ca_V_ channels, using immunohistochemical approaches, Grove et al. (2019) showed that antibodies against ρ-subunit-containing GABARs are expressed not in cones but rather in horizontal cells, and specifically in the cells’ synaptic endings where they would be localized close to the synaptic cleft (Figure 6). Super-resolution confocal images of retinal sections indicated co-localization of GABAR ρ2 subunits (and ρ1 subunits, not shown) with the horizontal cell marker calbindin in horizontal cell processes at synapses with both rods and cones(Grove et al., 2019). The structures show elements typical of electron microscopic images of horizontal cell invagination of photoreceptors and no evidence for ρ-subunit-containing GABARs in rods and cones. GABARs containing ρ-subunits have a high affinity for GABA and are non-desensitizing (Farrant and Kaila, 2007), consistent reports of a GABA-activated conductance in mammalian horizontal cells (Feigenspan and Weiler, 2004; Liu et al., 2013). The GABARs recently found in mouse cones (Deniz et al., 2019) do not include the ρ subunit-containing type shown here.

## The Cells That Release the GABA Respond to it: an Autaptic Mechanism

**Figure 7:**
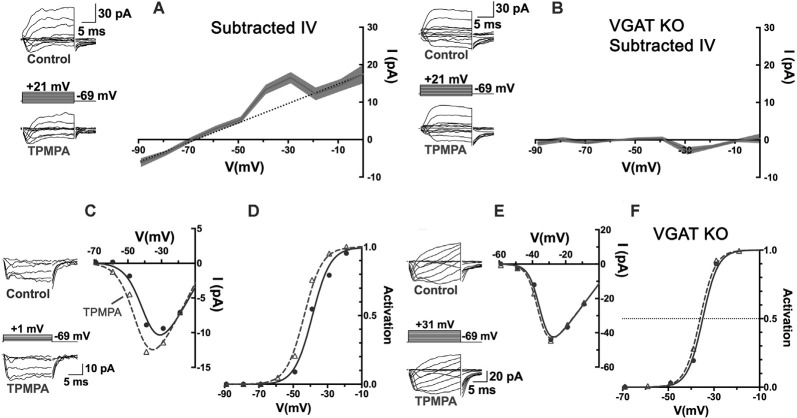
An autaptic mechanism: horizontal cells release GABA and respond to it via ρ-subunit-containing GABARs, and feedback to cones depends upon it. **(A)** Patch-clamp recording of Cx57-tdTomato labeled mouse horizontal cells in a slice bathed in CNQX (50 μM) and Hepes buffer (10 mM, pH 7.4) to isolate it from feedback and cone input. Whole-cell currents were elicited in horizontal cells with voltage steps, in control and in TPMPA (50 μM). Averaged I–V relations of steady-state TPMPA-subtracted currents showed a linear component reversing near −70 mV. Linear fitting excluded values near −40 and −30 mV. Gray shading shows the standard deviation of 5 horizontal cell recordings. **(B)** Same experiment and protocol as in panel **(A)** but recorded from Cx57-VGAT KO mouse horizontal cells that lack VGAT, which have synaptic vesicles devoid of GABA (Hirano et al., 2016). The mouse horizontal cells lacking VGAT were unaffected by TPMPA, suggesting that the tonic GABA levels in the wild-type are due to horizontal cell release and autaptic reception. **(C)** Recording of cone Ca_V_ channel currents in a wild-type mouse, showing current in control and in presence of TPMPA, with I–V relations. **(D)** Activation curves showed that the Ca_V_ channel activation midpoint shifted negative by about 5 mV in presence of TPMPA. Same experimental protocol as in panel **(C)** but in a Cx57-VGAT KO mouse cone, showing that the I–V relations **(E)** and the activation midpoints **(F)** were unaffected by TPMPA in cones when horizontal cells were unable to release GABA. Figure modified from Grove et al. (2019).

When currents in voltage-clamped horizontal cells, identified by their fluorescence in the Cx57-tdTomato mouse line (Hirano et al., 2016), were compared before and during the superfusion of TPMPA, the ρ-subunit-containing GABAR antagonist, a voltage-independent current reversing near the Cl^−^ equilibrium potential, was the only difference (Figure 7). The I–V relation obtained by subtracting the currents measured in TPMPA from those in control isolates the TPMPA-sensitive current, with a reversal potential near −70 mV. The recordings had to be performed in the presence of CNQX and Hepes to eliminate input from cones and possible effects of feedback. This mostly linear current component (notwithstanding the “bump” at −40 and −30 mV), being the current blocked by TPMPA, is by definition a ρ-subunit-containing GABAR current, revealed a tonic GABA-activated Cl^−^ current in the mouse horizontal cell.

The existence of a tonic GABA-activated current, that can be blocked with TPMPA over a broad range of potentials suggests that under the recording conditions used, GABA is tonically present in the synaptic cleft. This could arise from GABA being tonically released, even from modestly hyperpolarized horizontal cells, released from other GABAergic cells, or not being effectively removed from the synaptic cleft. As stated earlier, mammalian horizontal cells do not express GABA-uptake transporters, so there appears to be no localized removal mechanism in the protected synaptic cleft other than diffusion from the invagination followed by uptake by Müller cells (Bringmann et al., 2013). To test whether the tonic presence of GABA in the synaptic cleft is a result of horizontal cell release, horizontal cells incapable of expressing the vesicular GABA transporter, VGAT, were recorded from in Cx57-VGAT-KO mouse retinas (Hirano et al., 2016). In horizontal cells of this mouse line, the only retinal cell type expressing Cx57, the GABA transporter that loads synaptic vesicles with GABA is selectively deleted, rendering them incapable of releasing GABA. Matching the recording protocol used in Figure 7A but recording in VGAT-KO animals, TPMPA no longer caused any change in horizontal cell currents (Figure 7B). Horizontal cells in VGAT-KO mice exhibited normal expression of ρ-subunit containing GABARs in horizontal cells, so the fact that in the Cx57-VGAT-KO mice there was no TPMPA-sensitive current to be blocked means that in the control retinas, the horizontal cells respond autaptically to the GABA they release.

Returning to the outward current bump between −40 and −30 mV in Figure 7A, it is difficult to identify the source of this but it is possible since this is the range of voltages that peak Ca_V_ channel activation occurs in horizontal cells (Liu et al., 2016), that the bump reflects TPMPA block of extra GABAR Cl^−^ current arising from increased GABA release at those potentials. An increase of the horizontal cell Ca_V_ current due to alkalinization in the synaptic cleft, similar to that seen in cone Ca_V_ current when TPMPA is added (Figure 7C), could induce additional GABA release by the horizontal cell.

## The Tonic, Autaptic GABAR Current in Horizontal Cells Is Required for Feedback to Cones

Do the actions of TPMPA on cone Ca_V_ channels depend on the release of GABA from horizontal cells, which are the same cells that respond to it? Using the same Cx57-VGAT KO mice in which horizontal cell GABA release was eliminated, it was shown that the cone Ca_V_ currents, that underwent negative shifts in their activation midpoint in response to TPMPA in wild-type mice (Figures 7C,D), showed no change in their Ca_V_ channel activation in Cx57-VGAT-KO mice (Figures 7E,F). Since there is no evidence that there are functional ρ-subunit containing GABARs in cones or that those GABARs directly modulate Ca_V_ channel gating, the observed Ca_V_ channel activation curve shifts in cones were interpreted to be caused by GABA, released by horizontal cells and acting locally on horizontal cells via TPMPA-sensitive GABARs. However, this means that an additional “messenger” appears required to carry the signal from horizontal cells to the cone membrane where it affects cone Ca_V_ currents.

## The Effects of pH and GABA Merge Through a Concerted Biophysical Mechanism

The anion pore of GABA-activated channels is 20–60% as permeable to HCO_3_^−^ as it is to Cl^−^ (Bormann et al., 1987; Kaila and Voipio, 1987; Fatima-Shad and Barry, 1993; Hubner and Holthoff, 2013). A P_HCO3_/P_Cl_ of 0.29 was measured in rat horizontal cell GABAR channels (Liu et al., 2013). With internal and external HCO_3_^−^ concentrations both being in the 10–25 mM range, the considerable flux of this ion through GABAR channels has been shown to change extracellular pH in many brain areas (Bormann et al., 1987; Kaila and Voipio, 1987). This is how the pH sensitivity of the cone Ca_V_ channel activation is linked to GABA, being mediated by the flux of the two common permeant anions of GABA-activated channels, Cl^−^, and HCO_3_^−^, across the horizontal cell membrane in accounting for GABA-mediated inhibition and disinhibition of cone Ca_V_ channels, in the GABA-pH model (Liu et al., 2013; Grove et al., 2019).

The foundation of the GABA-pH hybrid model includes the following concerted biophysical mechanisms: (1) GABA acting on horizontal cell GABARs autaptically and tonically (Gilbertson et al., 1991; Kamermans and Werblin, 1992; Feigenspan and Weiler, 2004; Liu et al., 2013; Grove et al., 2019); (2) these GABARs mediating the efflux of the permeant anion HCO_3_^−^; and (3) the subsequent buffering of cleft pH, modulating photoreceptor transmitter release via surface charge effects on presynaptic cone Ca_V_ channels (Barnes et al., 1993; Vessey et al., 2005; Cadetti and Thoreson, 2006; Grove et al., 2019). The sign and magnitude of the contribution of the GABAR channel to cleft pH depend on the driving force on HCO_3_^−^, which is itself a pH-dependent function of the equilibrium potential for HCO_3_^−^ (E_HCO3_^−^), a value typically in the range of −10 to −20 mV (Roos and Boron, 1981), and the horizontal cell membrane potential.

Together, these concerted factors imply that, given a tonic presence of GABA in the synaptic cleft, disinhibition of cone Ca_V_ channels would be greatest when horizontal cell membrane potentials were most negative, thus producing more HCO_3_^−^ efflux due to the strengthened driving force. Whether reduced HCO_3_^−^ efflux at more positive horizontal cell voltages would be able to do the opposite, i.e., permit inhibition of photoreceptor Ca_V_ channels, due to the reduced alkalinizing influence, and otherwise allow acidifying influences to dominate, are questions addressed in the final section of this review.

## Horizontal Cell Depolarization Inhibits Photoreceptors

While HCO_3_^−^ efflux from horizontal cells during hyperpolarization accounts for the disinhibition of photoreceptor Ca_V_ channels caused by increased alkalinity, do horizontal cell depolarization produce inward flux of HCO_3_^−^ or reduce the outward driving force on HCO_3_^−^ efflux sufficiently to account for the *inhibition* of photoreceptor Ca_V_ channels? The outward rectification provided by BK channels (Sun et al., 2017), which activate steeply positive to −30 mV, prevents horizontal cell depolarization positive to E_HCO3_^−^, a value typically in the range of −15 to −20 mV for cells (Bolton and Vaughan-Jones, 1977). This means that an inward flux of bicarbonate would not occur under normal physiological conditions. But the reduced outward driving force on HCO_3_^−^ when the horizontal cell is depolarized, as occurs maximally in the dark, would lead to less pH buffering of the synaptic cleft and set the stage for acidifying influences to play an inhibitory role. What are those acidifying influences and how does activation of GABARs enhance this process?

**Figure 8:**
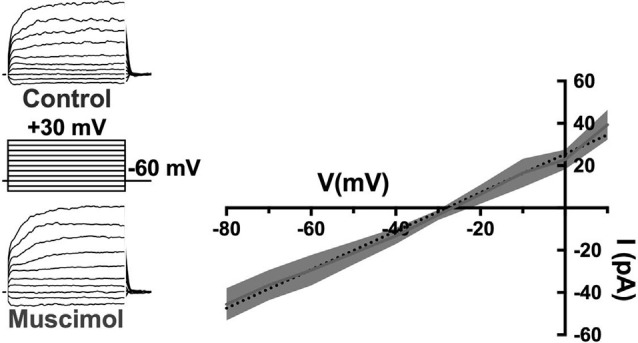
The GABA-agonist muscimol activates depolarizing currents in horizontal cells. Recordings of tdTomato-labeled mouse horizontal cells in slices produced currents in response to the voltage steps that were larger in the presence of 100 μM muscimol. Subtracting the control currents from those in muscimol produced a linear current-voltage relation reversing at −28 mV (I–V relation averaged from five horizontal cells; dotted line is a linear fit of mean subtracted currents, gray region indicates standard deviations). Gramicidin-perforated patch-clamp recording keeps intracellular [Cl^−^] intact, suggesting horizontal cells have a very elevated [Cl^−^] equilibrium potential as other studies have shown. Figure modified from Grove et al. (2019).

First, the activation of GABARs themselves contributes to the depolarization of horizontal cells. The GABAR agonist muscimol elicited currents reversing near −28 mV during recordings in mouse horizontal cells made with the gramicidin perforated patch technique, which preserves physiological intracellular chloride levels (Figure 8; Grove et al., 2019). This reversal potential of GABAR-activated currents suggested that E_Cl_ of the horizontal cell is much more positive than is typical for mature neurons, and has been previously observed for horizontal cells (Miller and Dacheux, 1983; Djamgoz and Laming, 1987; Kamermans and Werblin, 1992). Current-clamp recordings confirm that depolarization caused by GABAR activation contributes to positive membrane potentials in horizontal cells (Grove et al., 2019). Thus, GABAR activation depolarizes these cells, decreasing HCO_3_^−^ efflux, and this action could increase the acidifying influences on cleft pH.

**Figure 9:**
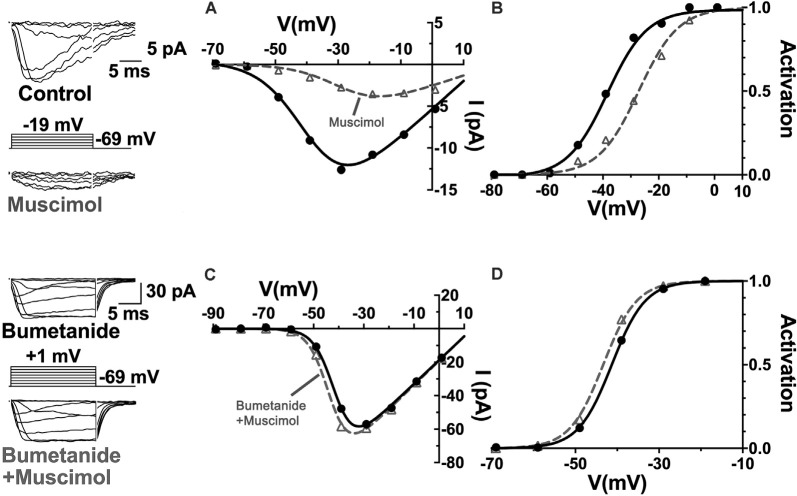
The NKCC blocker bumetanide eliminates the muscimol-induced activation curve shift of cone Ca_V_ channels. **(A)** I–V relations with smaller Ca_V_ channel currents were produced in the presence of muscimol, which is accounted for by the strong rightward shift of the Ca_V_ channel activation curve midpoint **(B)**. **(C)** Same experimental paradigm as above but slices superfused with bumetanide for 30 min. Ca_V_ channel currents in the absence and presence of muscimol produced I–V relations and activation curves having only a slight *leftward* activation midpoint shift with muscimol in bumetanide-treated retinas **(D)**. Figure modified from Grove et al. (2019).

Relatively positive equilibrium potential for Cl^−^ is produced in neurons by Na^+^/K^+^/Cl^−^ cotransporters (NKCC), that move Cl^−^ into cells electroneutrally using the Na^+^ and K^+^ gradients (Russell, 2000; Achilles et al., 2007). A specific subtype, NKCC1 (Slc12a2), has been previously identified in mammalian horizontal cells (Vardi et al., 2000) and would make any chloride conductance have a depolarizing effect. This depolarizing action of muscimol in horizontal cells (Figure 8) was shown to be due to a 7 mV positive shift of the Ca_V_ current activation curve (Figure 9; Grove et al., 2019). This outcome is consistent with the negative shifts of the cone Ca_V_ current activation curve produced by GABAR antagonists in Figures 4, 5, 7. When NKCC1 was blocked with bumetanide (Morita et al., 1999), the sign of muscimol’s cone Ca channel modulation was changed from inhibition to disinhibition. In the presence of bumetanide (50 μM), muscimol shifted the Ca_V_ current activation curve midpoint slightly negative (about 2 mV). This modest disinhibitory action induced by bumetanide could follow from the block of NKCC1 in horizontal cells, which would reduce intracellular [Cl^−^] to sufficiently low levels that GABAR activation no longer depolarizes them and might even produce hyperpolarization.

The disinhibitory action that followed the block of the inward Cl^−^transporter in horizontal cells suggests that the removal of the depolarizing influence of GABA allowed other actions, such as increased HCO_3_^−^ efflux, to become dominant and alkalinize the synaptic cleft, leading to the pH-dependent disinhibition of cone Ca_V_ channels. With tonic, the autaptic release of GABA by horizontal cells appearing to inhibit cone Ca_V_ channels in a manner due in part to its depolarizing effect on horizontal cells, the question remaining was what other cleft acidifying processes are initiated or increased when horizontal cells depolarize.

## Block of NHEs Produces Cleft Alkalinization Underlying Disinhibition of Cone Ca_V_ Channels

**Figure 10:**
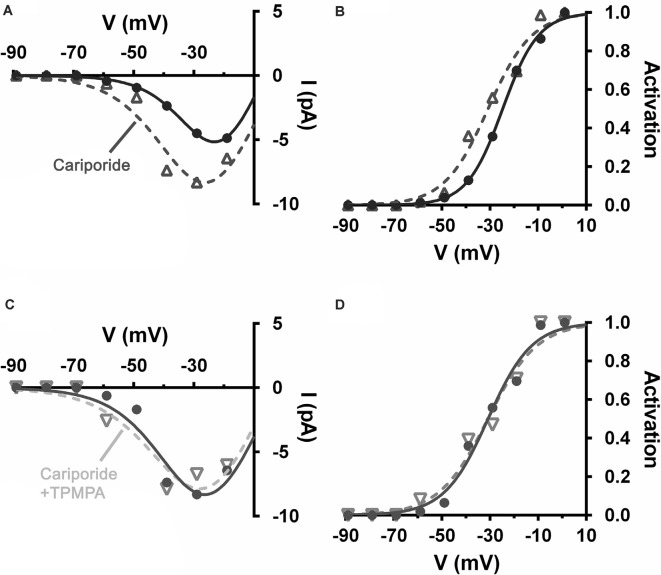
Alkalinization of the extracellular space produced by blocking Na^+^/H^+^ exchangers (NHEs), disinhibited cone Ca_V_ and occluded disinhibition by TPMPA. Recording of Ca_V_ currents in a mouse cone. **(A)** I–V relations show larger currents in the presence of cariporide (dashed line, open triangles) relative to control (solid line, filled circles). **(B)** The cone Ca_V_ activation curve shifts to a more negative potential following cariporide application (dashed line, open triangles). **(C)** In the same mouse cone bathed continuously with cariporide, I–V relations showed no effect of TPMPA on Ca_V_ current (dashed line, inverted triangles). **(D)** TPMPA failed to shift the Ca_V_ activation curve to more negative potentials (dashed line, inverted triangles).

NHEs were implicated in feedback inhibition of photoreceptors in non-mammalian vertebrates (Warren et al., 2016a). This acid extruder is electroneutral, meaning that it has no intrinsic voltage sensitivity and is not affected by membrane potential directly. However, NHEs are more active when neurons are depolarized due to their increased ion channel activity and the metabolic activity associated with ion pumping to restore gradients. Concomitant with this major influence, NHEs are sensitive to intracellular pH (pH_i_) and internal calcium levels and both of these stimuli increase with depolarization (Aronson et al., 1982; Madshus, 1988; Bertrand et al., 1994; Ma and Haddad, 1997; Koster et al., 2011). Increased NHE activity in mammalian horizontal cells is associated with horizontal cell modulation of cone Ca_V_ channels (Warren et al., 2016a; Grove et al., 2019). Using the same mesopic light-adapted retinal slices, the selective NHE blocker cariporide (10 μM), by itself, shifted mouse cone Ca_V_ channel activation curves negative by about 6 mV, disinhibition consistent with the block of NHEs that had had an acidifying effect on the synaptic cleft (Figures 10A,B). The sign and magnitude of this effect are close to that seen in Figures 4, 5 where the GABAR antagonist TPMPA was used by itself. Figures 10C,D show that the addition of TPMPA (50 μM) to the cariporide treated slice produced no shift of the Ca_V_ channel activation curve. Nearly identical results were reported using another blocker of NHEs, amiloride (30 μM), suggesting that the inhibitory effects of GABARs on cone Ca_V_ channel activation is due to conditions that NHE activity produced (Grove et al., 2019).

Figure 1 confirms that cone Ca_V_ channels are inhibited under steady-state mesopic conditions due to elevated NHE activity. The Block of NHEs leads to alkalinization of cleft pH, altering Ca_V_ channel gating like GABAR block with picrotoxin and TPMPA, shown in Figures 4, 5. However, an earlier report showed that GABA increased rat photoreceptor Ca_V_ currents under bright light, conditions under which horizontal cell membrane potential would be very negative (Liu et al., 2016), suggesting that GABA may therefore have alkalinizing effects when horizontal cells are hyperpolarized.

## GABARs and the Horizontal Cell Membrane Potential: Inhibition With Depolarization, Disinhibition With Hyperpolarization

The polarity of cone Ca_V_ channel modulation depends on the horizontal cell membrane potential.

**Figure 11:**
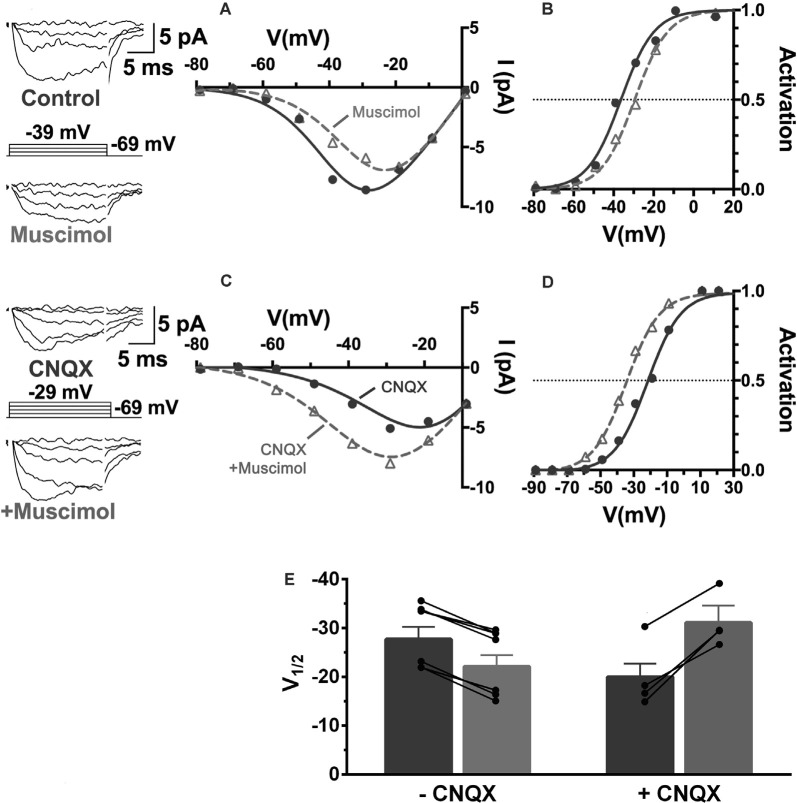
The sign of GABA’s influence on feedback is dependent on the horizontal cell membrane potential. Ca_V_ currents in cones are inhibited or disinhibited by muscimol depending on whether CNQX is present to block glutamatergic input to horizontal cells. **(A)** I–V relations show smaller cone Ca_V_ currents during muscimol superfusion in the absence of CNQX, due to Ca_V_ channel activation curve shifting 7 mV positive **(B)** during muscimol application. **(C)** In the presence of CNQX, which hyperpolarizes cones, muscimol shifted activation curve V_½_ to a more negative voltage **(D)**, leading to larger currents at all membrane potentials. **(E)** Changes to cone Ca_V_ channel activation curve midpoint caused by muscimol with or without the presence of CNQX show that the effect of muscimol depends on horizontal cell membrane potential as controlled by glutamate. Figure modified from Grove et al. (2019).

Recordings of the modulation of Ca_V_ channels in cones from retinas adapted to mesopic illumination and maintained in low light conditions showed that horizontal cells were in a relatively depolarized state (Grove et al., 2019). This is a well-known consequence of the release of glutamate by photoreceptors in low light. Horizontal cells are depolarized by glutamate, which is released in a graded manner by photoreceptors maximally in darkness. Horizontal cells rest under this condition at membrane potentials as high as −30 mV. It is broadly appreciated that reducing glutamatergic transmission with intense illumination or with glutamate receptor antagonists, horizontal cells hyperpolarize to levels near −60 mV (Hirasawa and Kaneko, 2003). When they are depolarized, horizontal cells inhibit photoreceptor Ca_V_ channels, and when they are hyperpolarized, they produce disinhibition of those channels (Thoreson et al., 2008). During recordings from cones in low light conditions with mesopic adaptation, as seen in Figures 9, 11A,B, muscimol (100 μM) application produced inhibition, a 6 mV rightward shift of the Ca_V_ channel activation curve (Grove et al., 2019). When retinas were superfused with CNQX (50 μM), which shifted cone Ca_V_ activation leftward 6 mV by itself, the effect of added muscimol application produced an 11 mV leftward shift in the cone Ca_V_ channel activation midpoint, a strong disinhibitory influence on the cone (Figures 11C,D). This result, summarized in Figure 11E, confirms earlier reports that photoreceptor Ca_V_ channel activation depends directly on horizontal cell membrane potential (Hirasawa and Kaneko, 2003; Cadetti and Thoreson, 2006; Babai and Thoreson, 2009; Grove et al., 2019), and that GABAR-mediated cone inhibition and disinhibition are functions of the horizontal cell membrane potential (Liu et al., 2013). This implies that the sign of GABA’s tonic influence on feedback depends on the immediate (light-dependent) polarization of the horizontal cell membrane potential.

NHEs never mediate H^+^ influx (Löscher et al., 2013) and therefore do not account for alkalization of the synaptic cleft in a hyperpolarized horizontal cell treated with CNQX. The Ca_V_ current disinhibition in cones seen in CNQX is due to horizontal cell GABAR-mediated HCO_3_^−^ efflux, which increases with horizontal cell hyperpolarization. GABAR activation with muscimol also failed to inhibit cone Ca_V_ channels in the presence of the NKCC1 blocker bumetanide and the NHE blockers amiloride and cariporide. These individual component effects support the conclusion that GABAR-mediated inhibition of cone Ca_V_ channels depends on horizontal cell depolarization and that this is outweighed by disinhibitory actions during hyperpolarization.

## Discussion and Conclusions

**Figure 12:**
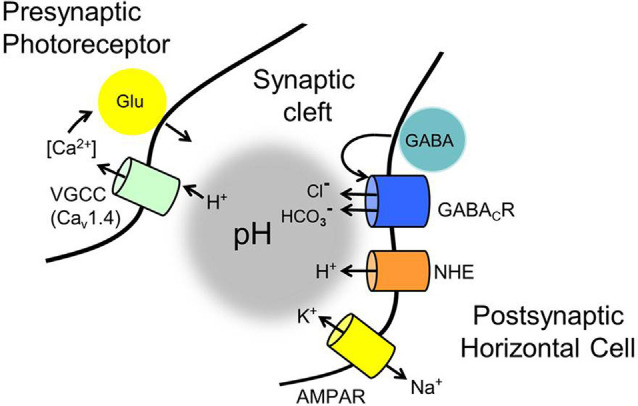
Membrane mechanisms identified in the GABA-pH hybrid model of feedback that have roles in modulating the pH of the mammalian photoreceptor synaptic cleft. Standing as the foundation of the feedback mechanism are voltage-gated Ca_V_1.4 channels in photoreceptors that ultimately regulate glutamate release. Notwithstanding ephaptic-coupled voltage changes of the cleft, never identified in mammalian retinas, regulation of the Ca_V_ channels by surface charge screening due to cleft pH changes has proven to be a powerful modulator of glutamate release. Contributors to cleft pH implicated in the current review include: (1) tonic autaptic release and reception of GABA by horizontal cells (only mammalian horizontal cells appear to release GABA via a vesicular mechanism, but an alternate mechanism, reversed GABA uptake, operates in many vertebrates). (2) While GABA release is controlled by horizontal cell Ca_V_ channels, evidence indicates that significant GABA levels are tonically present in the cleft, under light and dark-adapted conditions, and only change via membrane potential on longer time scales. (3) Tonic activation of ρ subunit containing GABARs provides HCO_3_^−^ efflux, whose amplitude is regulated by the driving force determined by the horizontal cell membrane potential, which is influenced strongly by photoreceptor glutamate release onto horizontal cell AMPARs, and the equilibrium potential for HCO_3_^−^. (4) HCO_3_^−^ efflux and depolarization-modulated proton extrusion via NHEs compete to change cleft pH. These steps result in the regulation of cone glutamate release in a manner dependent on horizontal cell *V*_m_ and this underlies the surround inhibition and regulation of synaptic strength, the hallmarks of horizontal cell feedback to cones.

This review integrates the guiding concepts about feedback that have emerged over the past half-century with findings from a recent report to describe a novel solution for how synaptic feedback occurs in mammalian retinas. To aid the comparison of this new GABA-pH hybrid model with earlier reports, Figure 12 summarizes the key membrane properties of photoreceptors and horizontal cells at the synaptic cleft that are central to the model. The central foundational tenet of the feedback mechanism is well-established in mammalian and non-mammalian vertebrates, namely, that horizontal cell depolarization inhibits photoreceptor voltage-gated Ca_V_ channels (Verweij et al., 1996). Two mechanisms for this channel modulation have been proposed, and both likely apply broadly, albeit with varying impacts upon the feedback process in different species. First, as described above, the electric feedback model (Byzov and Shura-Bura, 1986) and the hemichannel-mediated ephaptic coupling (Kamermans et al., 2001) posit that extracellular current flow into the synaptic invagination, terminating at the glutamate receptors and/or hemichannels at the tips of horizontal cell processes, changes the extracellular voltage, and this alters the photoreceptor Ca_V_ channel activation by changing the membrane electric field that governs channel gating. While compelling evidence for these actions comes from investigations in fish, none is available for connexion hemichannels at mammalian horizontal cell synaptic tips. Second, a host of reports show that feedback from horizontal cells is mediated by pH shifts within the synaptic cleft (Hirasawa and Kaneko, 2003; Vessey et al., 2005; Cadetti and Thoreson, 2006; Wang et al., 2014). Before this recent report (Grove et al., 2019), no convincing model of what drives the pH shifts as a function of horizontal cell membrane potential has emerged in mammals. The role of pannexin hemichannels in changing pH_o_ has been supported in zebrafish and invokes the efflux of ATP from horizontal cells through hemichannels followed by hydrolytic generation of phosphates capable of buffering pH (Vroman et al., 2014). The presence of pannexins is supported in fish (Cenedese et al., 2017), but the evidence does not support their appropriate localization in mammalian horizontal cells (Kranz et al., 2013). Although not considered a component of inhibitory feedback, the effect on Ca_V_ channel activation of a sudden pH reduction in the synaptic cleft was described during fast depolarization-induced vesicle fusion in photoreceptors, where the acidic vesicle contents are released to produce rapid and transient inhibition of the Ca_V_ channels responsible for the vesicle fusion (DeVries, 2001). Such an action, essentially due to a quick infusion of a bolus of protons, is fundamentally different than the continuous flow of protons via NHEs, or the continuously modulated efflux of the buffer HCO_3_^−^ described in this report.

Another mechanism involving the efflux of a pH buffer from horizontal cells, the GABA-pH model reviewed here in the *mammalian retina*, is more complex and implicates GABA in mediating the pH shifts. GABA that activates Cl^−^ and HCO_3_^−^ permeable GABAR autoreceptors are released tonically (Gilbertson et al., 1991; Kamermans and Werblin, 1992; Feigenspan and Weiler, 2004; Liu et al., 2013). In keeping with the tenet of feedback, i.e., that it is driven by the membrane potential of the horizontal cell, hyperpolarization is responsible for the tonic GABAR-mediated HCO_3_^−^ efflux that alkalinizes the synaptic cleft. It depends on the driving force on HCO_3_^−^, which is a function of the equilibrium potential for HCO_3_^−^ (E_HCO3_^−^; typically in the range of −15 to −20 mV) and the membrane potential of horizontal cells. This means that cone Ca_V_ channel disinhibition is greatest in the most negative range of horizontal cell membrane potentials, such as during stimulation with bright light. In contrast, the −30 mV reversal potential of GABAR can add to depolarization of the cell in a manner that increases cleft acidification, which is amplified by the non-linear H^+^ efflux via NHEs responding to intracellular metabolically driven acidification.

The results reported in Grove et al. (2019) and reviewed here are consistent with some features of the alternative hypotheses already presented in the literature. Often this is the case due to identities, or similarities, in the step-wise mechanisms, in non-mammalian and mammalian retinas. For example, whereas vesicular GABA release and its autaptic reception by horizontal cells is a key event in the GABA-pH model reviewed here, in fish and non-mammalian vertebrate retinas, the vesicular release seems to be replaced by reversed GAT-mediated uptake (Marc et al., 1978; Schwartz, 2002), which mammalian horizontal cells lack (Blanks and Roffler-Tarlov, 1982; Pow et al., 1996). The HCO_3_^−^ permeability of the GABARs should be biophysically conserved in all GABARs, requiring no species-specific claim. The pH-dependence of feedback is reported throughout studies in vertebrate retinas, and any change to the retinal environment that alters pH buffering or changes the pH is well-recognized as being capable of modulating the voltage-dependence of photoreceptor Ca_V_ channel activation. Hepes (and other pH buffers) block feedback in all models, suggesting a broadly consistent mechanism, albeit mediated by different mechanisms (pH buffering by HCO_3_^−^ in mammals vs. ATP hydrolysis in fish), but given a cytoplasm-acidifying alibi in the case of hemichannel ephaptic feedback (Vroman et al., 2014). The model reported in Grove et al. (2019) might not extend to all non-mammalian vertebrate species as some horizontal cell subtypes do not stain for GABA.

Feedback is considered to consist of several components (Warren et al., 2016b; Cenedese et al., 2017) that would function over different timescales. Analysis of the data of Grove et al. (2019) suggest three components, the slowest being the “tonic” presence of GABA in the OPL, which is expected to change on a scale of 10–100 min as the presence of GABA persists in the OPL. This component blends into the temporal range of and may be considered a part of, broader mechanisms of light and dark adaptation. A second, slow component is the depolarization-induced metabolic acidification of the OPL, operating on a scale of 10–100 s; and the GABA-pH model has a third and very fast component that involves membrane potential driven HCO_3_^−^ flux in GABAR channels, presumably in the millisecond range. Most of the experiments reviewed from Grove et al. (2019) studied feedback actions that involve all three components, with only the first and slowest generally held constant given the mesopic light-adapted conditions.

The speed of feedback is a very important issue and it has been argued effectively that there are fast and slow components in zebrafish. In zebrafish, the fast component is accounted for by the virtually instantaneous hemichannel-mediated ephaptic coupling, and the much slower component is caused by pannexin-mediated ATP release and resultant pH buffering action (Cenedese et al., 2017). In zebrafish, it has been suggested that GABA could also play a modulatory role in feedback, acting at the photoreceptor membrane, and switching feedback off during dark adaptation (Klaassen et al., 2011). Whereas the GABA-mediated modulation of feedback via cone GABARs is considered in zebrafish to be much slower than either of these (Klaassen et al., 2011), the GABA-pH model assigns GABA concentration as being essentially tonic, a condition that might be due to a low vesicular release rate (although still calcium-mediated) and the lack of uptake or degradation within the synaptic invagination (Grove et al., 2019). The existence of a constant HCO_3_^−^ semi-permeable membrane allows the pH-influencing effect in the cleft to be the fast component of feedback in this model, albeit it is somewhat slower than the truly instantaneous ephaptic coupling. It may be under-appreciated that the GABA-pH hybrid model attributes the tonic presence of GABA in the outer retina, experimentally confirmed in many of the figures in this review, as a gate for the presence of feedback. Once given this tonic GABA presence, changes to cleft pH are temporally linked to the speed of membrane potential change, as changes in horizontal cell membrane potential immediately change the driving force on HCO_3_^−^, followed by ion flux but not channel gating.

Some features of the different models are less amenable to interspecies conservation, a good example being the requirement for hemichannels in ephaptic feedback and their absence in mammalian retinas, suggesting that there is not hemichannel mediated feedback in mammalian retinas. This does not exclude AMPARs playing the traditional electric feedback role (Byzov and Shura-Bura, 1986). However, Warren et al. (2016b) carefully determined the instrument delay-corrected feedback timescales in salamander retina, finding the fastest time constant of feedback to be 9–13 and 116–216 ms for the slower component. A ~10 ms time constant is much slower than what is expected for an instantaneous ephaptic voltage change, discounting the traditional electric feedback by AMPARs (Byzov and Shura-Bura, 1986; Kamermans et al., 2001), but not a fast ion diffusion model.

### Dark-induced Acidification and Non-linear Proton Extrusion

The origin of dark-induced acidification in the OPL (Figure 2) lies with: (1) the sustained, depolarized state of photoreceptors, OFF-bipolar cells and horizontal cells in the dark; (2) their metabolic requirements to maintain ionic gradients under these conditions; and (3) the reactions by which mitochondria generate ATP. The metabolic demand arises principally from pumping ions by Na^+^/K^+^-ATPases, which utilize 1 ATP for every 3 Na^+^ pumped out in exchange for 2 K^+^ brought into the cell (Stahl, 1986), and to a lesser degree to cytosolic Ca^+^ extrusion by Ca^2+^-ATPases. The chemiosmotic theory holds that the synthesis of each ATP catalyzed by the H^+^-ATP synthase is coupled to the translocation of three to five protons depending on the type of synthase, from the internal mitochondrial compartment to the cytoplasm (Petersen et al., 2012) and, to maintain cytoplasmic pH, protons are extruded to the extracellular space principally via NHEs, producing extracellular acidification. When NHEs were blocked with cariporide or amiloride, inhibitory feedback was lost and there was no further regulation by TPMPA (Figure 10), providing evidence that NHE extrusion of protons into the extracellular space contributes significantly to extracellular acidification at the synaptic cleft (Warren et al., 2016a; Grove et al., 2019). This finding that horizontal cell GABAR-mediated cleft acidification is dependent on NHE proton extrusion supports a role for depolarization-mediated production and extrusion of protons in horizontal cell feedback. Although electroneutral, activation of the H^+^-extruder NHE is itself exponentially related to pH_i_ (Aronson et al., 1982) and NHE increases outward proton flux steeply due to its sensitivity to intracellular calcium and pH_i_ (Aronson et al., 1982; Madshus, 1988; Bertrand et al., 1994; Ma and Haddad, 1997; Koster et al., 2011). This causes perisynaptic NHE H^+^ efflux to increase exponentially with horizontal cell depolarization.

It is likely that, in low low-light conditions, depolarized photoreceptors, OFF bipolar cells, and horizontal cells contribute to this tonic acidifying influence that inhibits photoreceptor Ca_V_ channels. Since the role of the horizontal cell is to modulate photoreceptor output, horizontal cells limit this acidification by also introducing a pH buffer to the cleft pH as a function of their membrane potential. Cleft pH can rapidly increase during horizontal cell hyperpolarization due to voltage-driven efflux of HCO_3_^−^ via tonically active GABARs.

### GABA Was Previously and Erroneously Rejected as the Feedback Transmitter

Voltage clamp recordings of inward current induced by “pure” surround illumination upon the already standing spot response in mammals (Verweij et al., 2003) presents an outstanding example of the unintuitive nature of the actions underlying the GABA-pH hybrid model. In these experiments, a cone was stimulated with a small spot of light but was voltage-clamped at −40 mV, well within the activation range of cone Ca_v_, allowing glutamate release. The horizontal cell synaptic tips responding to the glutamate would be depolarized in these experiments. According to the GABA-pH hybrid model, GABA should decrease pH_o_ under these conditions. This is indeed what was found to occur; superfusion of GABA decreased feedback, while picrotoxin increased it. Surround-evoked inward currents persisted in picrotoxin and GABA, albeit with magnitudes that follow the shifted Cav activation curves shown here in cones voltage-clamped at −40 mV, and treated with picrotoxin and muscimol. Verweij et al.’s (2003) results do rule out a role for GABA *if* interpreted in the context that the GABARs would be on cones, not horizontal cells. The direct action of GABARs at the cone would have led to a block of surround-induced inward current changes when they applied either picrotoxin or GABA. Instead, the result presented is consistent with the autaptic GABA-pH feedback mechanism. Such an action is not predicted by any other of the existing models proposed for feedback and appears to reflect the full extent of the actions of feedback from horizontal cells to voltage-clamped cones, underscoring the differences seen in non-mammalian vertebrates, where cones express GABARs (Wu, 1991; Endeman et al., 2012).

The inconsistent effects of picrotoxin on horizontal cell feedback have been an additional confound in some earlier feedback models testing GABA as a potential transmitter. Studies that failed to find an effect with picrotoxin may have suffered from a late-emerging oversight. Due to the addition of a methionine residue in some species’ ρ2 subunits, their GABARs may be less sensitive to picrotoxin block (Zhang et al., 1995; Greka et al., 1998). The observation of GABAergic effects at relatively high concentrations of picrotoxin compared against those using standard concentrations of the selective ρ-subunit-containing GABAR antagonist TPMPA (50 μM), alongside mouse ρ2 GABAR immunostaining (Grove et al., 2019), suggest that studies testing the effects of picrotoxin using low concentrations could be compromised.

### GABA and pH Linked Actions Throughout the Brain

Changes in extracellular acidity in response to GABAergic activity have been described throughout the brain (Chesler, 2003; Farrant and Kaila, 2007; Ruusuvuori and Kaila, 2014). These are fundamentally similar to the GABA- and pH-mediated signaling in horizontal cell feedback but differ in detail. Most are mechanisms that share pH changing properties that affect synaptic acidification at GABA and glycinergic synapses elsewhere in the brain. In comparison, the GABA and pH-mediated effects in the horizontal cell to photoreceptor feedback represent a new form of synaptic inhibition in a graded potential network, and related pH-mediated modulations of synaptic interactions have been described in the CNS in action potential-dependent neurotransmission (Chesler, 2003; Farrant and Kaila, 2007; Ruusuvuori and Kaila, 2014).

In the olfactory bulb, signal processing requirements differ greatly from those in the eye. Synapses formed by olfactory receptors with periglomerular interneurons utilize GABAergic presynaptic feedback to modulate presynaptic Ca channels, albeit in a different manner than at photoreceptor synapses (McGann, 2013). This presynaptic inhibition has several roles, including the regulation of synaptic gain, and the generation of odorant filters that could sharpen olfactory discrimination. In the retina, inhibitory feedback to photoreceptors by horizontal cells functions to sharpen special contrast differences and to tune the response frequencies sensed by downstream retinal neurons, in a manner that sharpens temporal sensitivity.

The mechanisms reviewed here presented as changes to voltage-clamped Ca_V_ currents in cones and probed via the net effects of pharmacological manipulations, provide insight into steady-state levels of feedback. While all interventions that modulate the activation of the Ca_V_ current are not necessarily related to feedback from horizontal cells to photoreceptors, the dissection of steady-state conditions that effect feedback clarify in a stepwise manner the pathway that accounts for feedback. To fully appreciate this synaptic mechanism, all of these aspects should be considered simultaneously. A greater understanding of this novel feedback mechanism will be achieved with further testing using non-steady-state light conditions.

## Author Contributions

All authors: data curation, investigation, visualization, writing—review and editing. All authors contributed to the article and approved the submitted version.

## Conflict of Interest

The authors declare that the research was conducted in the absence of any commercial or financial relationships that could be construed as a potential conflict of interest.
